# Exploring the Practice of Dual Vaping: Health Risks and Behavioral Patterns in Nicotine and Cannabis E-Cigarette Users

**DOI:** 10.3390/brainsci15020097

**Published:** 2025-01-21

**Authors:** Giovanna Nelda Vaccari Bongetta, Raony Ferreira França, Maria Olivia Pozzolo Pedro, Kae Leopoldo, Luiza Larrubia Alvares Florence, Israel Kanaan Blaas, Gislaine Koch Gimenes, Julio Torales, Antonio Ventriglio, Domenico de Berardis, João Mauricio Castaldelli-Maia

**Affiliations:** 1 Department of Neuroscience, Medical School, FMABC University Center, Santo André 09060-870, Brazil; ginelda19@gmail.com (G.N.V.B.); israel_blaas@hotmail.com (I.K.B.); jmcmaia2@gmail.com (J.M.C.-M.); 2 Department of Psychiatry, Medical School, University of São Paulo, São Paulo 05403-903, Brazil; raonyffranca@gmail.com (R.F.F.); maria.oliviapozzolo@gmail.com (M.O.P.P.); kae.leopoldo@usp.br (K.L.); gislaine.koch@hc.fm.usp.br (G.K.G.); 3 Department of Preventive Medicine, Federal University of São Paulo, São Paulo 01246-903, Brazil; luiza.florence@yahoo.com; 4 Instituto Perdizes (IPER), Hospital das Clinicas HCFMUSP, Faculdade de Medicina, Universidade de Sao Paulo, São Paulo 05021-001, Brazil; 5 Facultad de Ciencias Médicas, Cátedra de Psicología Médica, Universidad Nacional de Asunción, San Lorenzo 111421, Paraguay; juliotorales@gmail.com; 6 Facultad de Ciencias de la Salud, Universidad Sudamericana, Salto del Guairá 140101, Paraguay; 7 Department of Clinical and Experimental Medicine, University of Foggia, 71121 Foggia, Italy; dr.ventriglio@gmail.com; 8 Department of Mental Health, ASL4 Teramo, 64100 Teramo, Italy; 9 Pharmacology, School of Nursing, University of L’Aquila, 67100 L’Aquila, Italy; 10 International Centre for Education and Research in Neuropsychiatry, University of Samara, 443100 Samara, Russia

**Keywords:** electronic nicotine delivery systems, marijuana smoking, tobacco use, vaping

## Abstract

Background: E-cigarettes, initially designed for nicotine consumption, are now increasingly being used to smoke cannabis, resulting in a growing trend known as “dual vaping”. This term describes individuals, referred to as “dual users”, who use e-cigarettes for both substances. This study aims to review and analyze existing research on dual vaping, with a focus on the associated health risks and behavioral patterns. Methods: A narrative review of the literature was conducted using PubMed. Studies focusing on individuals who use electronic cigarettes for vaping tobacco and cannabis, either chronically or episodically, were examined. Relevant articles were identified and thematically synthesized to provide a comprehensive overview of the topic. Results: Dual vaping was found to be prevalent among younger men, White and Hispanic populations, and individuals with higher economic status and educational level. The use of one substance was shown to predispose individuals to the use of the other, often leading to concurrent use of both substances. Peer influence and positive expectations regarding e-cigarettes were identified as significant predictors of dual use. Dual vapers exhibited a higher susceptibility to respiratory and systemic symptoms compared to those who exclusively vaped nicotine or cannabis. Moreover, a notable prevalence of psychiatric disorders, such as substance use disorders, anxiety, and depression, was observed in this group. Fruit-flavored e-cigarettes were the most preferred option among dual vapers when using both nicotine and cannabis. Conclusions: Current evidence is insufficient to fully elucidate the long-term impacts of dual vaping on physical and mental health, particularly when compared to individuals who have never vaped. Further studies are needed to gain a more comprehensive understanding of this behavior.

## 1. Introduction

Electronic cigarettes (also known as e-cigarettes, e-cigs, vapes) were first developed in China in 2003 and later disseminated to other Western countries with the aim of aiding cigarette users in smoking cessation. However, recent meta-analyses have shown limited effectiveness of e-cigarettes for this purpose and have also raised concerns about their safety [1,2]. Most electronic cigarettes vaporize a mixture known as “e-liquid” or “juice”, which typically contains nicotine, glycerine or propylene glycol, and a flavoring agent [3]. The use of the devices for smoking cannabis has gained popularity, leading to a significant rise in individuals who use vapes for both cannabis and tobacco, a practice referred to as dual vaping [3,4]. Understanding the implications of this dual use is critical for assessing its public health impact and guiding effective prevention strategies.

Since their introduction on the market, electronic cigarettes have undergone several modifications in their appearance, design, battery power, atomizers, and nicotine delivery systems. Today, four generations are recognized [5,6]: (a) devices shaped like a traditional cigarette, featuring an atomizer that produces aerosol by heating the juice, thereby preventing tobacco from combustion; (b) pen-style devices with an atomizer and a larger tank compared to the first generation; (c) devices with even larger tanks, adjustable batteries, allowing voltage and power variation by the user; (d) the latest and most popular models, which are refillable. These advancements have increased the versatility of e-cigarettes, further expanding their use for substances beyond nicotine.

A survey conducted among university students in the United States (US) revealed that the majority of individuals who used e-cigarettes to vaporize a substance other than nicotine reported using cannabis or its derivatives [3]. The addition of fragrant flavors enables users to discreetly smoke tetrahydrocannabinol (THC), masking the characteristic odor of traditional cannabis cigarettes [7]. This compound is often consumed through vapes in the form of hash oil (a highly concentrated D-9-tetracannabidiol) or dried cannabis buds and leaves [8]. In a single session, users can inhale up to 50 mg of THC, compared to approximately 12 mg delivered by a typical marijuana joint [9]. Rapid THC delivery and higher quantities increase the risk of hallucinations, psychosis, cannabinoid hyperemesis syndrome, as well as mental health and behavioral disorders [9]. These findings underscore the importance of exploring the health risks associated with dual vaping practices.

There is sufficient evidence supporting the bidirectional association between tobacco smoking and cannabis smoking [10,11]. According to Mattingly et al., this association is also confirmed between nicotine and cannabis vaping [12]. This interplay may be due to the proximity of endocannabinoid and nicotine receptors in key areas of the reward system, such as the midbrain, hippocampus, and amygdala [13]. Although most individuals vape nicotine and cannabis separately, the practice of mixing these substances in the same liquid has been growing, especially among young people [12]. Combined use is associated with a greater risk of respiratory discomfort, physical and mental health problems, increased cannabis dependence, and greater difficulty in quitting both substances when compared to using just one [4,11]. This highlights a critical gap in understanding the behavioral and physiological drivers behind dual vaping.

THC vaping is strongly associated with the development of the E-cigarette or Vaping Use-Associated Lung Injury (EVALI), a clinical condition responsible for a public health crisis in the US in 2020, resulting in the hospitalization of 2807 people with 68 deaths [14]. One potential causal agent of the EVALI is vitamin E acetate, used as a thickening agent in homemade THC e-liquid sold on the black market [15]. Most patients with EVALI report respiratory symptoms, such as cough, dyspnea, and chest pain, along with gastrointestinal and constitutional symptoms [16]. Computed tomography typically reveals bilateral ground-glass opacities in the lungs, with pneumomediastinum, pleural effusion, and pneumothorax reported as possible complications [17]. E-cigarettes may also alter the human immune system by decreasing B lymphocyte counts and inducing a pro-inflammatory state in the airways, increasing the risk of respiratory infections [18]. A similar pro-inflammatory state may occur in the perioral region, leading to changes in the oral microbiota and deterioration of periodontal, dental, and gingival health [19]. These findings further underline the urgent need for targeted public health interventions to mitigate the risks of dual vaping.

The vaporization of e-liquid produces fine particulate matter, similar to tobacco-combustion products, potentially increasing the risk of platelet aggregation and cardiovascular diseases [20]. Furthermore, research evidence suggests harmful effects of vaping on endothelial function and arterial thickness, which increase the long-term risk of coronary events [21]. During vaporization, trace metals that are potential carcinogens, such as nickel, chromium, cadmium, aluminum, and lead, can leach from the atomizer into the e-liquid and be inhaled [22]. However, as this technology is relatively recent, there is no confirmed evidence yet linking electronic cigarette use to the development of cancer. Despite these uncertainties, the cumulative evidence points to significant health risks associated with e-cigarettes, particularly among dual users, underscoring the importance of continued research and regulation in this area.

The increasing use of electronic cigarettes, particularly for dual vaping of tobacco and cannabis, presents emerging health risks that are not yet fully understood. This review aims to synthesize the current evidence on dual vaping, focusing on its health implications, the role of vaping in both tobacco and cannabis consumption, and the demographic factors that predispose individuals to dual use. By addressing these issues, this study contributes to a better understanding of the public health risks associated with dual vaping and provides direction for future research and policy development.

## 2. Materials and Methods

This narrative review was conducted using PubMed as the primary database for identifying relevant studies. The search focused on the literature related to dual vaping of tobacco and cannabis and was performed up to August 2023. The search terms included combinations of keywords such as “cannabis”, “marijuana”, “nicotine”, “vape”, and “e-cigarettes”, applying Boolean operators to refine the results and capture relevant studies addressing the dual use of these substances. Studies were included if they focused on the use of electronic cigarettes for vaping both nicotine and cannabis, examined either chronic or episodic use, and were published in English. Exclusion criteria encompassed studies conducted in vitro or with animals, editorials, commentaries, and case reports, as well as articles that did not explicitly address dual vaping. Titles and abstracts of identified studies were screened for relevance, and full-text articles were retrieved for detailed evaluation when necessary. Articles that met the inclusion criteria were then analyzed and categorized into key themes based on their findings, including user demographics, health risks, behavioral patterns, and predisposing factors for dual vaping. A thematic synthesis approach was employed to summarize and integrate the findings, highlighting commonalities and differences across the selected studies.

## 3. Results

The characteristics of the studies included in this narrative review are presented in Table 1. Most studies were carried out in the US, with combined sample size of 424,116 participants, ranging in age from 12 to 86 years. All studies were published between 2019 and 2023.

### 3.1. Demographics of Users

Uddin et al. (2020) analyzed the differences between three groups of vapers: dual (nicotine and cannabis), predominantly nicotine, and predominantly cannabis users [40]. They found an association between dual vaping and increasing age compared to nicotine-predominant vaping (dual users = 17.2% aged 18–24 years, 19.7% aged 45–69 years; nicotine-predominant users = 33.2% aged 18–24 years, 23.7% aged 45–69 years). Most users across all the three categories were men (58.5% in the dual use group), had an educational background, and reported a family income above the poverty line. Among dual vapers, the majority were White (54.0%), followed by Hispanics (27.1%), Black individuals (14.9%), and other groups (4.00%).

Watson et al. (2020) analyzed 3980 dual vapers and, in contrast, found a higher prevalence of women (53.5%) with an average age of 36 years. The majority of their sample (28.8%) was aged between 25 and 34 years [26]. However, they reported a high prevalence of White participants (71.0%), followed by Hispanics (11.0%) and Black individuals (10.0%).

Young-Wolff et al. (2021) studied 363 adolescents aged 12–17 years in Northern California and found that 40% were dual vapers [31]. Most were male (66.7%), and the most prevalent ethnicities were non-Hispanic Whites (53.7%), Hispanics (23.1%), and Asians and Pacific Islanders (19.0%). Adolescents with a higher family income were more likely to use vaping (USD 80,000–120,000, aORs = 2.05–3.34; >USD 120 K, aORs = 3.68–9.48) compared to those with a family income of less than 80,000 dollars.

Saran et al. (2022) conducted an online survey with 503 vape-users in the US and identified 357 dual users [28]. Among these, men were predominant (63.2%), and most were Whites (87.4%). This study reported that 78.4% of dual users were married or living with a partner, 81.1% identified as heterosexual, 77.5% reported a bachelor’s degree or higher, 90.2% held a full-time job, and 83.7% were current cigarette smokers.

### 3.2. EVALI

Werner et al. (2020) studied the reports of EVALI submitted to the Center for Disease Control and Prevention (CDC) up to January 2020, which included 60 fatal and 2558 non-fatal cases [23]. Non-exclusive THC vapers accounted for 67% of fatal cases and 79% of non-fatal cases. Non-exclusive nicotine vapers represented 56% and 55%, respectively. Dual vapers represented 27% of fatal cases and 40% of non-fatal cases. Finally, 4% and 6% of cases involved individuals who did not vape any substances.

Blount et al. (2020) analyzed bronchoalveolar fluid from 51 hospitalized patients with a confirmed or probable diagnosis of EVALI. Among them, 77% reported vaping cannabis, 67% nicotine, and 51% both substances [25]. A higher prevalence of THC vaping among EVALI patients (86%, n = 1604) was observed in the report by Moritz et al. (2019) [42]. Other studies have reported somewhat conflicting results regarding the higher prevalence of EVALI between cannabis-only vapers and dual vapers.

Lewis (2019) examined 53 EVALI reports submitted to the Utah Department of Health [33]. Of those, 92% vaped THC, 66% nicotine, and 60% both. Layden et al. (2020) reported that all patients with EVALI in their study (n = 98) had vaped in the previous 90 days (a finding also noted in a smaller study with only 8 participants) [36], with 92% vaping in the preceding week [24]. Of the 81 patients interviewed, 27% vaped only THC, 11% only nicotine, and 60% both. Kligerman et al. (2021), after diagnosing 160 patients with EVALI according to the computed tomography findings, found that 48.1% vaped only THC, 9.4% only nicotine, and 42.5% both substances [32]. Interestingly, a significant correlation was observed between the substance vaped and the presence of documented fever: 68.8% of the cannabis-only vapers had fever, compared to 66.2% of dual vapers and 33.3% of nicotine-only vapers. Despite this difference, the presence of fever did not affect the severity of the lung injury.

### 3.3. Other Health Consequences

Smith et al. (2022) [30] surveyed 112 individuals who reported dual vaping within the past 30 days, utilizing the Global Assessment of Individual Needs Short Screener (GAIN-SS) scale [30]. They observed that the majority demonstrated a moderate to high risk of internalizing problems (mean ± standard deviation: 3.00 ± 1.60), externalizing problems (1.80 ± 1.50), and substance use disorder (2.00 ± 1.50). Other studies have similarly identified a higher prevalence of cannabis dependence and nicotine dependence among dual vapers compared to those using only one substance [28]. Additionally, a high prevalence of substance use disorder involving alcohol, opioids, cocaine, and hallucinogens, for instance, as well as depression and anxiety, was reported [31].

Case et al. (2022) used data from the Texas Adolescent Tobacco and Marketing Surveillance System to describe symptoms related to the e-cigarette use [16]. Compared to non-users, dual vapers and nicotine vapers reported a higher risk of respiratory symptoms such as cough, chest pain, wheezing, and shortness of breath, relative to cannabis vapers (aOR = 2.35, 95% CI: 1.30, 4.25; AOR = 1.86, 95% CI: 1.22, 2.81, respectively). Regarding gastrointestinal symptoms, cannabis-only vapers reported a higher prevalence (AOR = 2.41, 95% CI: 1.53, 3.79), followed by the nicotine-only group (AOR = 2.03, 95% CI: 1.38, 2.99), and dual vapers (AOR = 2.03, 95% CI: 1.15, 3.57). Overall, the risk of constitutional symptoms, including headache, appetite changes, dehydration, tiredness, fever, and weight fluctuations was highest among dual vapers, followed by the nicotine-only and cannabis-only groups.

### 3.4. Behaviors

Moustafa et al. (2022) conducted a prospective cohort study using questionnaires with ninth graders to identify the pattern of cannabis and nicotine vaping use and users’ characteristics [27]. Compared to non-users, dual vapers were more likely to have used cigarettes (OR = 2.39, 95% CI = 1.05, 5.45) and alcohol (OR = 4.39, 95% CI = 2.71, 7.11) in the last six months. They also reported higher odds of peer vaping (OR = 1.20, 95% CI = 1.08, 1.34), sensation-seeking (OR = 1.11, 95% CI = 1.07, 1.15), and positive e-cigarette expectations (OR = 1.17, 95% CI = 1.10, 1.24). Positive e-cigarette expectations were also observed in the study published by Bessenyei et al. (2023) (OR = 1.05, 95% CI: 1.01–1.10, *p* = 0.022) [46].

In the report by Dugas et al. (2020), nicotine-only vapers were more likely to report a lifetime quitting attempt (77.1%, n = 35) than dual users (62.5%, n = 8) [41]. However, more dual vapers (37.5%) perceived that e-cigarettes helped them quit compared to the nicotine-only group (28.6%). Conflicted results were found in Saran et al. [28], where dual users, when compared to nicotine-only vapers, were more likely to have tried quitting. The dual group has also begun vaping nicotine at a younger age, used higher nicotine concentrations, was more likely to buy their nicotine vape online or from friends/family than from a gas station, and was less likely to use nicotine vape as a replacement for combustible cigarettes. Compared to cannabis-only vapers, dual vapers started using cannabis vape at a younger age, were more likely to endorse having a state medical marijuana card and purchase their vape online or from friends/family than from a dispensary.

Watson et al. (2020) collected data from an online survey involving 3980 adults aged ≥ 18 years [26]. They reported using THC- and nicotine-containing electronic vaping products (EVPs). In the previous three months, a high percentage also smoked marijuana (90.1%) and conventional cigarettes (63%). Additionally, this study evaluated the most common flavors preferred, with fruit (71%), candy (39.4%), mint (35.2%), and menthol (34.3%) being the top preferences for THC-containing EVP, and fruit (59.9), menthol (45.6), mint (37.3), and tobacco (34.2) for nicotine-containing EVP.

### 3.5. Predisposing Factors

Taleb et al. (2020) showed that, compared to non-users, the use of e-cigarettes (aOR = 1.67, 95% CI = 1.32, 2.11) was associated with marijuana vaping [34]. Participants who believed e-cigarettes were “equally addictive” to cigarettes were less likely (aOR = 0.79, 95% CI = 0.65, 0.97) to ever vape marijuana than those who considered e-cigarettes less addictive than cigarettes. Furthermore, the odds of vaping marijuana increased in a dose-dependent manner as the lifetime frequency of e-cigarette use increased. Compared to participants who used e-cigarettes for 10 days or less in their lifetime, those who used them for 11 to 50 days (aOR = 1.61, 95% CI = 1.22, 2.13), 51 to 100 days (aOR = 2.13, 95% CI = 1.41, 3.22), and more than 100 days (aOR = 2.69, 95% CI = 1.89, 3.82) were more likely to have ever vaped marijuana. A significant positive association between e-cigarette use and marijuana vaping was also found in many other studies [29,35,37,38,39,43,44,45], with odds ratios varying from 2.16 (95% CI = 1.20, 3.89) in Lee et al. (2021) [39] to 19.76 (95% CI = 17.29, 22.57) in Keyes et al. (2022) report [29]. Furthermore, in the latter study, it was reported that those who vaped and smoked nicotine were more than 40 times likely to vape and smoke cannabis. In the study by Baldassarri et al. (2020), an association between nicotine and cannabis vaping was only seen for adults aged 25 to 54 years (aOR = 4.6, 95% CI = 2.70, 7.78) [38]. Among youths aged 18 to 24 years, such an association was not confirmed (aOR = 0.9, 95% CI = 0.33, 2.26).

## 4. Conclusions

This narrative review highlights the increasing prevalence of dual vaping, particularly among younger individuals and those with higher socioeconomic status. Given the rising prevalence of this behavior, further research is needed to explore key areas. First, studies should aim to include a wider range of populations to examine how dual vaping impacts various demographic groups. This could help identify differences across age, gender, ethnicity, and socioeconomic status, expanding our understanding of who is most at risk.

Additionally, while EVALI has been primarily studied in the United States, it is essential to expand research globally to understand the broader implications of dual vaping. Long-term health outcomes, such as respiratory issues [14,15,16,17], systemic inflammation, and psychiatric disorders [28,30,31], should be investigated in diverse cultural and healthcare settings to assess global trends. Given the compounded health risks associated with dual vaping, future research should also focus on the long-term effects of using both tobacco and cannabis through e-cigarettes.

Research should also delve deeper into the behavioral patterns associated with dual vaping, including the role of peer influence, social norms, and the psychological mechanisms that drive dual use. Additionally, exploring the bidirectional relationship between nicotine and cannabis use may shed light on shared addiction pathways. The preference for flavored e-cigarettes, particularly fruit flavors, highlights the need for studies on the role of flavor in initiating and sustaining dual vaping. Understanding how flavors influence adolescent and adult users’ choices could inform regulatory efforts to limit their appeal to younger demographics.

Furthermore, future studies should focus on developing targeted prevention and intervention strategies for dual vapers. Research should examine effective regulatory measures, public health campaigns, and educational programs aimed at reducing dual vaping, especially among high-risk groups such as younger individuals and those with existing substance use disorders. Given the complex interplay between tobacco, cannabis, and alcohol use, future studies should adopt interdisciplinary approaches, incorporating insights from public health, psychology, and pharmacology. This will help deepen our understanding of how these substances interact at the physiological level and the combined effects they have on the brain’s reward systems, which may contribute to addiction and sustained dual use.

## Figures and Tables

**Table 1:** Summary of studies included in the narrative review on dual vaping.

Author, Year	Type of Study	Study Population (n, Age)	Methods	Results	Limitations
Werner et al. (2020) [23]	Observational	n = 2618 cases of EVALI Age = 15–75	A national study comparing the characteristics of patients with fatal and non-fatal cases of EVALI	THC Use: Non-exclusive THC-containing product use was reported by 67% of fatal cases and 79% of non-fatal cases. Nicotine Use: Non-exclusive nicotine-containing product use was reported by 56% of fatal cases and 55% of non-fatal cases. Dual Use: Both THC- and nicotine-containing products were used by 27% of fatal cases and 40% of non-fatal cases. No Use: Neither THC nor nicotine use was reported by 4% of fatal cases and 6% of non-fatal cases.	Low fatal case numbers, potential misclassification of substance use, and ascertainment bias due to reliance on proxy interviews for deceased patients.
Layden et al. (2019) [24]	Observational	n = 98 cases of possible EVALI Age = 14–30	An interview was administered to characterize the use of e-cigarettes in the 3 months before symptom onset.	Respiratory symptoms: 97% had symptoms, with 85% experiencing shortness of breath and cough, and 52% reporting chest pain. E-cigarette use: All patients used e-cigarettes or related products within 90 days, and 92% of those with data vaped the week before symptoms; most (69/78) used daily. Substance use: 27% used THC only, 11% nicotine only, and 60% used both.	The study relied on patient-reported exposure information, which may be subject to recall bias, and likely captured more severe EVALI cases, potentially overlooking milder presentations.
Blount et al. (2019) [25]	Observational	n = 51 cases of EVALI and 99 healthy individuals Age = 21–45	Bronchoalveolar lavage (BAL) fluids were collected and analyzed using isotope dilution mass spectrometry to accurately measure the presence of various toxicants.	Vitamin E acetate: Found in 94% of case patients (48/51) but absent in the healthy group. THC or metabolites: Detected in 94% of case patients (47/50) or linked to reported THC vaping within 90 days. Nicotine or metabolites: Found in 64% of case patients (30/47).	Due to limited BAL fluid volume, not all analytes were tested for all participants. Sample collection was non-standardized as part of routine clinical care, and exposure timing and levels relative to sample collection were not evaluated.
Watson et al. (2022) [26]	Observational	n = 3980 Age = 19–86	Data were collected through adults who reported using THC- and nicotine-containing vapes in the past 3 months.	Demographics: 53.5% female; median age 36. Ethnicity: 71% non-Hispanic White, 11% Hispanic. Substance use: 90% used marijuana in the past 3 months; 62.6% smoked cigarettes.	The study relied on self-reported product use, introducing potential recall bias, included data from only 18 U.S. states, had a predominantly non-Hispanic White sample, and did not differentiate between medical and non-medical THC use.
Moustafa et al. (2022) [27]	Prospective longitudinal	n = 1835 9th-grade adolescents Age = nan	A survey on nicotine and cannabis vaping was conducted in four public high schools, repeated every six months over 36 months.	The sample was divided into four groups: early, declining dual use (Class 1), rapidly increasing dual use (Class 2), later, slower dual use (Class 3), and no use (Class 4). Class 1 and Class 2 (vs. Class 4): Associated with cigarette, cigar, and alcohol use, peer vaping, sensation-seeking, and positive e-cigarette expectations. Class 3 (vs. Class 4): Associated with alcohol use, positive e-cigarette expectations, depressive symptoms, and sensation-seeking.	Substance use was self-reported and may be subject to recall bias. The results may not generalize nationally, especially in states with different cannabis policies.
Saran et al. (2022) [28]	Observational	n = 503 adult nicotine and/or cannabis vapers Age ≥ 21	Data of dual vs. nicotine-only and cannabis-only vape users were compared.	Final sample: 357 dual vapers, 40 cannabis-only, and 106 nicotine-only vape users. Dual vapers started vaping younger, used it for more years, and were less likely to use nicotine vapes to replace cigarettes. Dual users did not have significantly higher nicotine or cannabis dependence scores compared to single-substance users.	A convenience sample that was primarily male, White, and college-educated was used.
Keyes et al. (2022) [29]	Observational	n = 51,052 Age: nan	A survey assessed past 30-day cannabis use, analyzing trends by sex, race/ethnicity, parental education, and urbanicity.	Past 30-day frequent and occasional cannabis vaping rose from 2017 to 2019. Hispanic/Latinos and adolescents with lower socioeconomic status experienced notable increases in frequent cannabis vaping. Adolescents who reported 10+ occasions of binge drinking and the ones who reported smoking and vaping nicotine were 10 and 42 times more likely to report past 30-day cannabis vaping, respectively, compared with no use.	The questionnaire lacked data on cannabis quantity, assessed binge drinking uniformly at five or more drinks, included only school-attending adolescents, and, being cross-sectional, could not establish causality.
Smith et al. (2022) [30]	Observational	n = 112 current vapers Age: 18–65	A survey was used to assess inhaled modes of nicotine and cannabis.	Participants who vaped nicotine and cannabis monthly also reported monthly smoking of cannabis (100%), and cigarettes (58%). Most exhibited moderate-to-high degrees of mental health and substance use problems requiring clinical intervention.	The study used a small convenience sample and cross-sectional data, preventing causal attributions between nicotine and cannabis vaping and smoking.
Young-Wolff et al. (2021) [31]	Observational	n = 363 Age = 12–17	Participants underwent addiction intake evaluations, and multivariable logistic regression tested associations between socio-demographics, cigarette smoking, substance use disorders, and vaping behaviors.	Majority of adolescents reported ever (68%) or current (60%) vaping nicotine and/or cannabis. Current vaping rates were similar for nicotine (50%) and cannabis (51%), with 40% reporting dual vaping. Black adolescents had lower odds of current nicotine and dual vaping. Alcohol use disorder was linked to current vaping. Believing most friends get drunk/high increased odds of current cannabis vaping. Higher income and never vs. ever blunt use were associated with higher odds of all vaping outcomes.	Participants had access to a large healthcare system. The cross-sectional design prevents causal attributions between vaping and substance use, and self-reported outcomes may involve recall bias.
Kligerman et al. (2021) [32]	Observational	n = 160 EVALI cases Age = 15–68	CT scans were analyzed to correlate imaging findings, pattern frequencies, and injury severity with substances vaped, vaping frequency, geography, and state THC legality. Statistical methods included one-way ANOVA, χ^2^ tests, and multivariable analyses.	48.1% of patients reported vaping THC, 9.4% nicotine, and 42.5% both. Common imaging findings included diffuse or lower lobe ground-glass opacification (78.1%) with subpleural (78.1%), lobular (59.4%), or peribronchovascular sparing (40%), septal thickening (50.6%), lymphadenopathy (63.1%), and consolidative nodules (36.3%). Increased vaping frequency was associated with more severe lung injury.	EVALI is a diagnosis of exclusion, potentially overlooking undiagnosed infections or inflammatory conditions. Most cases lacked pathological confirmation.
Lewis (2019) [33]	Observational	n = 83 EVALI reports Age = 14–66	Characteristics of medical care, potentially related conditions, and exposures were described.	Among 53 interviewed patients, all of whom reported using e-cigarettes within 3 months of acute lung injury 49 (92%) reported using products containing THC, 35 (66%) nicotine-containing products, and 32 (60%) both. Among all 83 patients, 69 (83%) were male. The most common self-reported comorbidities were anxiety (34%), depression (23%), and asthma (20%).	Non-interviewed patients (36%) and Utah’s THC illegality may have led to under-reporting of use and pre-existing conditions. Reporting bias is possible due to data primarily from pulmonologists and critical care physicians.
Ben Taleb et al. (2020) [34]	Observational	n = 10,680 middle- and high-schoolers Age: nan	Cross-sectional analysis of the 2018 National Youth Tobacco Survey (NYTS). A multivariable regression model was conducted to assess factors associated with vaping marijuana.	Overall, 26% of participants reported ever vaping marijuana. High schoolers (vs. middle-schoolers), Hispanics, and Black individuals were more likely to ever vape marijuana. Those who perceived e-cigarettes as equally addictive to cigarettes were less likely to ever vape marijuana. Those who reported ever trying cigarettes, cigars, or hookah were more likely to ever vape marijuana.	The survey only assessed ever vaping marijuana, missing frequency or current use, and illegal marijuana use in many states may have led to under-reporting.
Boakye et al. (2021) [35]	Observational	n = 160,209 US adults Age ≥ 18	Prevalence and trends of past-30-day cannabis vaping, with multivariable logistic regression analyzing associations with high-risk behaviors and diseases.	Past-30-day cannabis vaping prevalence increased from 1% to 2% from 2017 to 2019, with the greatest increase among young adults, from 1.2% to 3.9%. Cannabis vaping was associated with higher odds of binge drinking and other high-risk behaviors.	Data were self-reported, potentially introducing recall bias. Only the primary method of cannabis use was reported, possibly underestimating vaping, and data were limited to nine U.S. states.
Case et al. (2022) [16]	Observational	n = 2389 adolescents Age = 17–24	Data analyzed associations between past 30-day vape user categories and 15 health symptoms using statistical methods.	For total symptoms, dual vapers reported the highest mean, followed by nicotine-only vapers, marijuana-only vapers, and never users. Dual vapers and nicotine-only vapers had significantly higher odds of experiencing respiratory symptoms as compared with never users. Dual vapers had higher odds of having respiratory and gastrointestinal symptoms compared with never users.	Since it is a cross-sectional study, vaping cannot be confirmed as the cause of the described symptoms.
Pray et al. (2020) [36]	Observational	n = 8 confirmed or probable EVALI cases Age = 16–20	The study linked tetrahydrocannabinol (THC)-containing products and vitamin E acetate to the condition through interviews, laboratory analysis of vaping products, and bronchoalveolar lavage (BAL) fluid analysis.	All 8 patients reported daily use of THC-containing e-cigarettes in the month preceding the symptoms. All patients reported daily use of nicotine-containing e-cigarettes. Vitamin E acetate was detected in all five THC cartridges tested from two patients, and in BAL fluid from two other patients.	The small sample size, delayed data collection (4 months post-symptom onset), and limited testing of THC cartridges and BAL fluids (only half the sample) may introduce recall and selection bias.
Smith et al. (2021) [37]	Observationa	n = 12,064 Age = 16–19	Weighted multivariable regression models assessed correlates of co-use and seven cannabis delivery methods.	Dual use prevalence: 8.8% in Canada, 9.7% in the U.S., and 7.6% in England. 39.9% of past 30-day nicotine users reported cannabis use, while 69.7% of cannabis users reported nicotine use. Co-use among nicotine users was associated with ever alcohol use and lower harm perceptions of smoked cannabis. Co-users of cannabis had higher odds of reporting depression symptoms.	The cross-sectional design and potential under-reporting of cannabis due to its illicit status in some areas.
Baldassarri et al. (2020) [38]	Observational	n = 8255 individuals who recently used marijuana Age = 18–64	Regressions adjusted for demographics analyzed associations between marijuana vaping and medical marijuana use, conventional cigarette use, and nicotine e-cigarette use.	The odds of marijuana vaping were higher among those who reported using it for medical purposes and lower among people who smoked combustible cigarettes. Vaping nicotine e-cigarettes was associated with greater odds of vaping marijuana for adults aged 25–54 years but not for those aged 18–24 years.	Data were self-reported, potentially introducing bias, with possible under-reporting of marijuana use in states where it is illegal. Only 15 U.S. states were included.
Lee et al. (2021) [39]	Observational	n = 7821 Age = 12–17	The study used multivariable logistic regression to examine marijuana vaping initiation at Wave 4 among non-users at Wave 3, based on key youth substance use risk factors.	Marijuana vaping initiation is associated with the current use of electronic nicotine delivery systems, cigarettes, other marijuana products, and alcohol. Marijuana vaping initiation include being 15–17 years old, Hispanic, having less than college-level parental education, peer vaping, and a high internalizing and externalizing tendency.	Data are self-reported and subject to recall bias. Since the study is cross-sectional, no causal inference can be established. Marijuana use might be under-reported in states where it is illegal.
Uddin et al. (2020) [40]	Observationa	n = 131,807 Age ≥ 18	Data on past 30-day marijuana use were used to describe the emerging dual nicotine and marijuana vaping population.	Nicotine-predominant, marijuana-predominant, and dual vaping was 3.36%, 1.09%, and 0.38%, respectively. Dual and marijuana-predominant vapers were older, had higher proportions of non-Whites (especially Hispanics), and were less likely to be current smokers compared to nicotine-predominant vapers. Dual vapers among e-cigarette users: 8.6% in 2016, 2.6% in 2017, and 7.1% in 2018.	Self-reported data may introduce recall bias, and the cross-sectional design prevents causal conclusions.
Dugas et al. (2020) [41]	Observational	n = 775 young adult Age = nan	Data from a longitudinal study on cigarette smoking and nicotine dependence were collected using mailed self-report questionnaires.	19% of participants reported past-year e-cigarette use. Among e-cigarette users, 55% vaped cannabis, 50% vaped nicotine, and 39% used nicotine-free e-liquid. 82% also used other nicotine products, including 72% who smoked conventional cigarettes. 60% reported nicotine dependence symptoms, rising to 79% among nicotine vapers. 29% tried quitting cigarettes with e-cigarettes, but only 16% found them helpful.	The sample of e-cigarette users was small. The nicotine content of e-liquid is often mislabeled, which might have led to under-reporting.
Moritz et al. (2019) [42]	Observational	n = 1378 EVALI reports Age = 13–75	Data were used to describe patient characteristics, substances used in e-cigarettes, and characteristics of EVALI-associated deaths.	Median age: 23 years for surviving EVALI patients, 45 years for those who died. Among 867 patients with e-cigarette data, 86% used THC products, 64% used nicotine products, and 52% used both in the 3 months before symptoms. Among 19 patients who died, 84% used THC products, and 37% used nicotine products.	Self-reported or proxy data may have recall bias and misclassification due to varying collection methods across states. Marijuana’s illegality in many states likely led to under-reporting.
Lanza et al. (2020) [43]	Prospective Cohort	n = 3322 high school’s students Age ≥ 18	Students were surveyed on trajectories of nicotine and cannabis vaping at 6-month intervals from the fall of 11th grade to the spring of 12th grade, and again 1 to 2 years after high school.	Most participants were non-users (67.6% for nicotine, 64.9% for cannabis), followed by infrequent users (17% nicotine, 18.3% cannabis), and smaller proportions were moderate users (5% nicotine, 7% cannabis) or frequent users. Polysubstance vaping was common, with frequent nicotine vapers having a high likelihood of also engaging in cannabis use.	The sample was geographically limited, and data on substance use were self-reported, making it subject to recall bias.
Rychert et al. (2023) [44]	Observational	n = 23,500 Age ≥ 16	An online survey used multivariate logistic regression models to identify predictors of daily vaping of nicotine, non-nicotine e-liquids, cannabis e-liquids/oils, and cannabis herb.	42% of past 6-month vapers used a vaporizing device daily or near-daily. Nicotine was the most common substance used by daily vapers (96%), followed by dry herb cannabis (12%), no-nicotine e-liquids (10%), and cannabis e-liquid (6%). Cannabis use frequency was negatively correlated with daily nicotine vaping and positively correlated with daily vaping of no-nicotine and cannabis. Younger age predicted daily vaping of nicotine and no-nicotine liquids, while older age predicted daily cannabis vaping.	A convenience sample was used. Data on substance use were self-reported, and subject to recall bias. As the study is cross-sectional, no causal associations can be made.
Worthen et al. (2023) [45]	Observational	n = 339 undergraduate students Age = 18–35	A cross-sectional survey was conducted among undergraduate students to examine ENDS or vaping use, including substances used, reasons for initiation, and perceptions of harm and regulation.	49% reported using ENDS or vaping in the past 30 days. The most commonly vaped substances were cannabis (34%), nicotine (26%), and flavor (19%). Reasons for initiating ENDS use were social (64%), for the high (40%), and for the flavor (32%). Both users and non-users believed ENDS were harmful and favored regulation.	Self-reported data may introduce recall bias, and the non-probability sampling limits generalizability. ENDS use may be underestimated due to the over-representation of health-related students.
Bessenyei and Yakovenko (2023) [46]	Observational	n = 615 cannabis adult vapers Age = 18–30	Hierarchical binary logistic regression was employed to predict whether participants belonged to a polysubstance vaping group or a single-substance vaping group.	23% reported dual vaping, 25% cannabis-only, and 51% nicotine-only. None of the demographic variables showed a significant difference between the polysubstance and the single substance user group. Higher impulsivity, more substances used, and male gender were associated with an increased likelihood of dual vaping.	Some important factors, like social media influence, were not measured, and predictions of vaping intention do not guarantee actual behavior.
